# Genetic Inactivation of *Trpml3* Does Not Lead to Hearing and Vestibular Impairment in Mice

**DOI:** 10.1371/journal.pone.0014317

**Published:** 2010-12-13

**Authors:** Simone Jörs, Christian Grimm, Lars Becker, Stefan Heller

**Affiliations:** Departments of Otolaryngology – Head and Neck Surgery and Molecular and Cellular Physiology, Stanford University School of Medicine, Stanford, California, United States of America; Duke University, United States of America

## Abstract

TRPML3, a member of the transient receptor potential (TRP) family, is an inwardly rectifying, non-selective Ca^2+^-permeable cation channel that is regulated by extracytosolic Na^+^ and H^+^ and can be activated by a variety of small molecules. The severe auditory and vestibular phenotype of the TRPML3(A419P) varitint-waddler mutation made this protein particularly interesting for inner ear biology. To elucidate the physiological role of murine TRPML3, we conditionally inactivated *Trpml3* in mice. Surprisingly, lack of functional TRPML3 did not lead to circling behavior, balance impairment or hearing loss.

## Introduction

The mammalian TRPML gene family consists of TRPML1, TRPML2, and TRPML3. Mutations in human TRPML1 cause the lysosomal storage disease Mucolipidosis Type IV [1]–[3]. The murine *Trpml2* and *Trpml3* genes were identified in a positional cloning approach to find the gene responsible for the varitint-waddler (*Va*) phenotype [4]. Heterozygote *Va* mice have pigmentation defects, hearing loss, and circling behavior, whereas homozygotes display perinatal lethality [4]–[6]. The *Va* phenotype is caused by a single mutation (A419P) in the predicted fifth transmembrane-spanning domain (TM5) of TRPML3, which leads to a constitutively open channel, resulting in elevated [Ca^2+^]_i_ causing apoptosis [7]–[11]. Heterologously expressed TRPML3 is regulated by extracytosolic Na^+^ and H^+^ [8], [12], [13] and can be activated by a variety of small molecules [13]. Subcellular localization studies of heterologously expressed TRPML channels revealed that TRPML1 and TRPML2 are expressed in late endosomes and lysosomes, whereas TRPML3 presumably shuttles between multiple intracellular compartments and the plasma membrane [14]. The localization to intracellular vesicles and the interaction with a variety of vesicular proteins suggest that all three members may play a role in endocytic and exocytic signalling pathways [15]. In addition, heterologously expressed TRPML protein subunits are able to heteromerize with each other [13], [16], and it has been hypothesized that plasma membrane-localized TRPML3 in epidermal melanocytes occurs exclusively as a subunit of uncharacterized heteromeric channels [13].

TRPML3 expression has been detected in inner ear sensory hair cells [4], [17], and the *Va* mutation of the channel leads to hair cell degeneration and deafness [4], [5]. On the other hand, hair cell death-mediated deafness (due to a constitutive active ion channel) is not sufficient to justify a function in the hearing process itself. To elucidate a relevant function for TRPML3 in hair cells, we generated and examined a mouse model that allows conditional *Trpml3* inactivation. Here we show that ubiquitous as well as hair cell-specific postnatal inactivation of *Trpml3* does not result in hearing and balance impairment when compared with control littermates.

## Results and Discussion

### Generation of a floxed Trpml3 allele (*Trpml3^lox^*)

**Figure 1:**
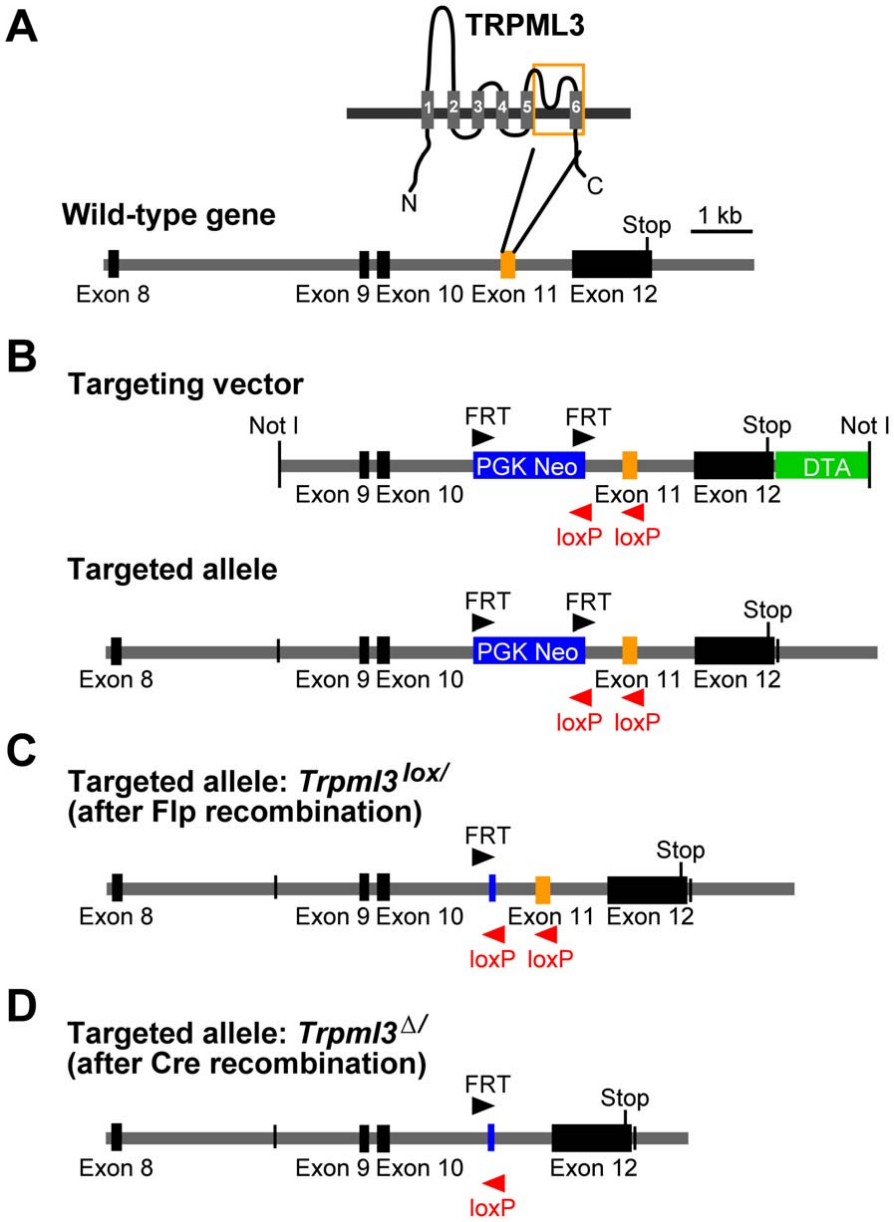
Targeting strategy for disruption of the *Trpml3* gene. A, Transmembrane-spanning domains are depicted as grey bars and numbered from 1–6. The orange frame indicates the part of the TRPML3 protein that is encoded by exon 11, which will be deleted (pore loop and TM6). Exons are shown as black and orange bars on the schematic genomic map below. B, Shown are the targeting vector and the targeted allele after homologous recombination. The blue bar represents the PGK promoter-driven *neo^R^* expression cassette, which was used for positive selection. The DTA cassette, used for negative selection, is shown in green. The black and red arrowheads symbolize position and orientation of *FRT* and *loxP* sites. C, Targeted allele after Flp site-specific recombination in ES cells, resulting in excision of *neo^R^* cassette, and leaving one FRT site behind. D, Excision of exon 11 using Cre site-specific recombinase, resulting in disruption of *Trpml3* gene.

**Figure 2:**
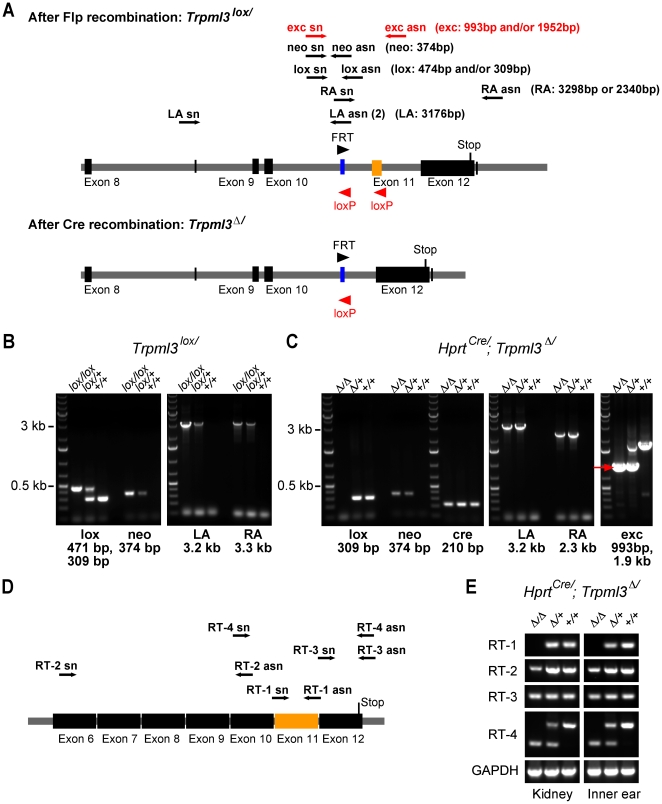
Genotyping analysis. A, Schematic drawing of the *Trpml3* targeted allele before (*Trpml3^lox/^*) and after Cre recombination (*Trpml3 ^Δ/^*). The positions of sense (sn) and antisense (asn) oligonucleotides are indicated with arrows. The names of PCR products and the corresponding lengths are parenthesized. B, PCR amplification products of *Trpml3* locus of homozygous (lox/lox), heterozygous (lox/+), and wild-type (+/+) *Trpml3^lox/^* mice are shown: lox: 309 bp and/or 471 bp, neo: 374 bp, and left arm (LA): 3176 bp and right arm (RA): 3298 bp. C, Genotyping after Cre recombination of representative *Hprt^Cre/^;Trpml3 ^Δ/^* mice. All mice are heterozygous for *cre* (210 bp). The same sets of oligonucleotides were used as in (B), however lox-PCR only displayed the wild-type 309 bp PCR fragment, since the lox asn-oligonucleotides cannot hybridize after the targeted exon 11 was excised; and RA: 2340 bp, since exon 11 was excised by Cre. The last panel on the right shows the shortened fragment (indicated with a red arrow): 1952 bp for *Trpml3^lox/^* and 993 bp for *Trpml3 ^Δ/^*. D and E, RT-PCR analysis of TRPML3 mRNA expression in kidney and inner ear from 3-week-old *Hprt^Cre/^;Trpml3 ^Δ/^ mice*. D, Schematic illustration is showing exon 6–12. The sense (sn) and antisense (asn) oligonucleotides are indicated with black arrows. E, Amplification products RT-1 (200 bp), RT-2 (512 bp), RT-3 (181 bp), and RT-4 (473 bp for wild-type cDNA, and 266 bp for Cre targeted *Trpml3* cDNA). Oligonucleotides for GAPDH (442 bp) were used to control for RNA preparation quality. The symbol Δindicates the deleted exon 11 in the *Trpml3* allele generated by recombination of the floxed (*loxP*) locus; +, represents the wild-type locus.

TRP channel knockouts have successfully been generated through targeted deletion of a genomic region encoding the presumptive pore-loop domain of the ion channel [18]–[20]. Therefore, we decided to target exon 11 of *Trpml3*, which encodes the pore-loop and the pore lining TM6 (Fig. 1A). We generated a targeting vector that, after homologous recombination, resulted in a modified *Trpml3* allele carrying two *loxP* sites flanking exon 11 (Fig. 1B). A *neo^R^* cassette, which was used for G418/geneticin selection, was removed with Flp recombinase before the ES cells were injected into host blastocysts to generate chimeric mice (Fig. 1C). After germline transmission and continued breeding, PCR with genomic DNA from progeny of wild-type, heterozygous and homozygous animals showed proper recombination and inheritance of the *Trpml3^lox^* allele (Fig. 2A,B). Exon 11 of the *Trpml3^lox^* allele and the genomic sequences surrounding the recombination site were verified by sequencing.

### Excision of *Trpml3* exon 11

To investigate whether TRPML3 plays a role in development, we used the X-linked *Hprt^Cre/+^* driver, which mediates ubiquitous and highly efficient excision that is complete at the first stages of development [21]. *Hprt^Cre/+^*;*Trpml3^lox/+^* females were mated with *Trpml3^lox/+^* males. The floxed exon 11 was always excised regardless of *Hprt^Cre^* inheritance. Oocytes of *Hprt^Cre^* females have sufficiently stored Cre recombinase to excise floxed DNA segments already at the zygote or early stages [21], which most likely resulted in *Trpml3* inactivation already in the fertilized egg. The resulting *Trpml3 ^Δ^* mutation was stably inherited (Fig 2C–E).

To examine whether the *Trpml3 ^Δ^* allele is being transcribed, RT-PCRs were conducted (Fig. 2D,E). mRNA from kidney and inner ear was purified from dissected organs of mice of each genotype. RT-PCR amplification of specific cDNA sequences before and after the site of deletion indicated that wild-type and *Trpml3 ^Δ^* mRNA was expressed in kidney and the inner ear of all animals (Fig. 2D,E; RT-2 and 3). Amplification of cDNA encoding exon 11 (RT-1), however, was only possible from mRNA of wild-type (*Trpml3^+/+^*) and heterozygous animals (*Trpml3 ^Δ/+^*), but not from mRNA of *Trpml3 ^Δ/Δ^* mice.

**Figure 3:**
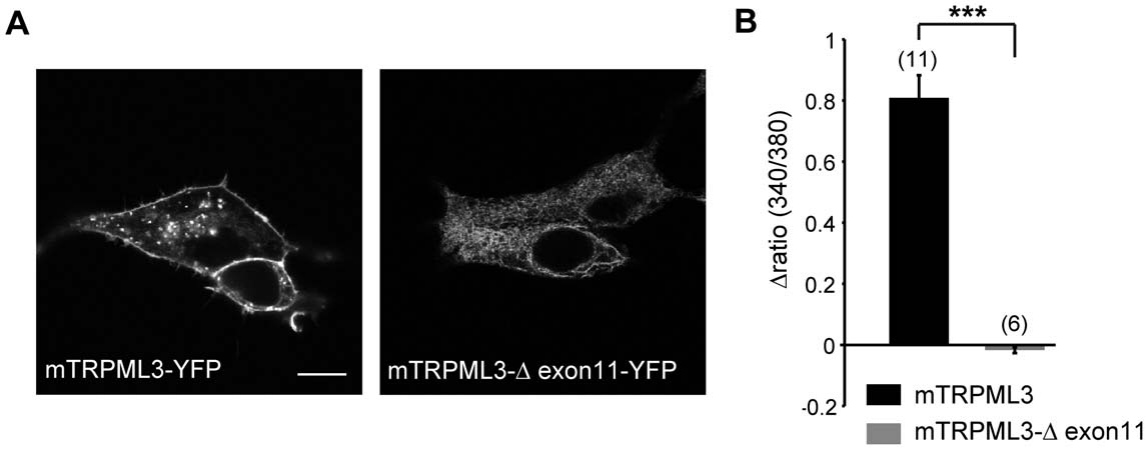
Molecular and functional assessment of mutant TRPML3(Δ exon11) channels. A, Shown are representative cells expressing the respective murine (m) constructs of wild-type TRPML3 or mutant TRPML3(Δ exon11), C-terminally fused to yellow fluorescent protein, 24 h after transfection. Scale bar = 10 µm. B, Ca^2+^ imaging results showing relative [Ca^2+^]_i_ increases after application of TRPML3 activator SN-2 in HEK293 cells expressing wild-type TRPML3 or mutant TRPML3(Δ exon11). Shown are mean values ± SEM (numbers in parentheses are the numbers of independent experiments with 10–20 cells each). Statistical comparisons of means were made using Student's *t* test (unpaired); ***p<0.0001.

An additional oligonucleotide pair was used (RT-4) to amplify the coding region comprising exons 10, 11, and 12. Sequence analysis revealed that in mutated animals, splicing occurred between exon 10 and exon 12, resulting in a 207 bp shorter message encoding a protein lacking 69 amino acids of the pore loop and TM6, but without frame shift. To demonstrate that a pore-less TRPML3 protein does not function as an ion channel, we generated cDNA encoding a fusion protein of TRPML3(*Δ* exon11) with yellow fluorescent protein and expressed the mutant channel protein in HEK293 cells. The subcellular distribution of the mutant channel TRPML3(*Δ* exon11), analyzed by confocal microscopy, was different from wild-type TRPML3-YFP fusion protein. Mutant channel protein appeared to be absent from the plasma membrane and was mainly located intracellularly (Fig. 3A). When we used the TRPML3 agonist SN-2 to activate TRPML3(*Δ* exon11) [13], we detected no change of [Ca^2+^]_i_, whereas wild-type TRPML3-expressing cells responded with a robust increase in [Ca^2+^]_i_ (Fig. 3B). The average maximum [Ca^2+^]_i_ levels were 0.81±0.073 (Δratio 340nm/380nm, n = 10) for wild-type TRPML3 and −0.016±0.009 (Δratio 340nm/380nm, n = 6) for TRPML3(Δ exon11); the latter measurements did not differ significantly from the baseline average ratio of 0.319±0.038 (n = 6), indicating that TRPML3(*Δ* exon11) is an inactive ion channel when expressed in HEK293 cells. Based on these experiments, we presume that *Trpml3 ^Δ/Δ^* mice lack functional TRPML3 protein.

### Evaluation of Balance and Auditory Function

Based on the *Va* phenotype, TRPML3 has been proposed to play a role in hearing and balance and the channel has been put forward as potential candidate for the hair cell mechanoelectrical transduction channel [22]. Consequently, we were curious to find out whether *Trpml3* inactivation would result in hearing and balance defects. Three-week-old *Hprt^Cre/+^*;*Trpml3^+/+^*, *Hprt^Cre/+^*;*Trpml3 ^Δ/+^*, and *Hprt^Cre/+^*;*Trpml3 ^Δ/Δ^* mice had normal Preyer's reflexes, characterized by a distinct movement of the pinna in response to a loud sound [23].

Auditory-evoked brainstem response (ABR) measurements were used to evaluate hearing thresholds of three-week-old mice (Fig. 3). Because the Preyer's reflex and other subjective measures to assess hearing are only effective for identification of profound hearing loss, more objective electroacoustical tests were conducted: Click, 8-, 16-, and 32 kHz tone burst measurements revealed no significant differences in ABR thresholds and interwave latencies among all three groups: *Hprt^Cre/+^*;*Trpml3^+/+^*, *Hprt^Cre/+^*;*Trpml3 ^Δ/+^*, and *Hprt^Cre/+^*;*Trpml3 ^Δ/Δ^* (Fig. 3A,B). Interwave latencies between wave I and wave III at 70 dB, which are indicative of the afferent auditory nerve conductance were 1.9±0.026 msec in *Hprt^Cre/+^*;*Trpml3^+/+^* animals (n = 6), compared to 1.87±0.026 msec in *Hprt^Cre/+^*;*Trpml3 ^Δ/+^* (n = 6), and 1.86±0.019 msec in *Hprt^Cre/+^*;*Trpml3 ^Δ/Δ^* littermates (n = 6). These results suggested that TRPML3 is not essential for hair cell function, synaptic signal transmission, performance of spiral ganglion neurons, and auditory nerve function. Nevertheless, because the inactivation of *Trpml3* using *Hprt^Cre^*–mediated recombination was introduced very early in development, compensation effects cannot be excluded with this approach.

With the goal to circumvent potential compensatory mechanisms, we used tamoxifen-inducible *Math1-Cre*ER™ mice [24] to inactivate TRPML3 in cochlear hair cells between P0–P3. ABR measurements of these mice at three weeks of age revealed no differences (Fig. 3C), suggesting that compensatory mechanisms most likely do not explain the lack of a TRPML3 hearing phenotype. Mice were also analyzed at 3 months of age to determine possible enhancement or early onset of age-related hearing loss. But reviewing audiograms revealed no ABR threshold difference between mutant mice and control mice (data not shown). These experiments have two important limitations. First, the tamoxifen-inducible recombination in cochlear hair cells is not complete, as revealed by analysis of crosses of *Math1-Cre*ER™ mice with *Rosa26-lacZ* reporter mice [24]. Therefore, a substantial number of cochlear hair cells might have been unaffected by Cre-mediated recombination. The fact that mutant TRPML3 protein is being expressed (Fig. 3A) complicates the analysis of Cre-mediated recombination because the mutant pore-less TRPML3 protein is likely to be detectable by immunohistochemistry. Second, three weeks of loss of TRPML3 function might be enough for potential compensatory mechanisms to become effective. Nevertheless, as long as there are no clear candidates or mechanisms known that could provide compensation for inactivation of *Trpml3*, it is difficult to speculate about the timing of compensatory mechanisms. In summary, the use of *Math1-Cre*ER™ mice did not reveal potential compensatory mechanisms of *Trpml3* inactivation, but because of the limitations of this experiment, we cannot exclude that loss of TRPML3 function is being compensated by an unknown mechanism.

**Figure 4:**
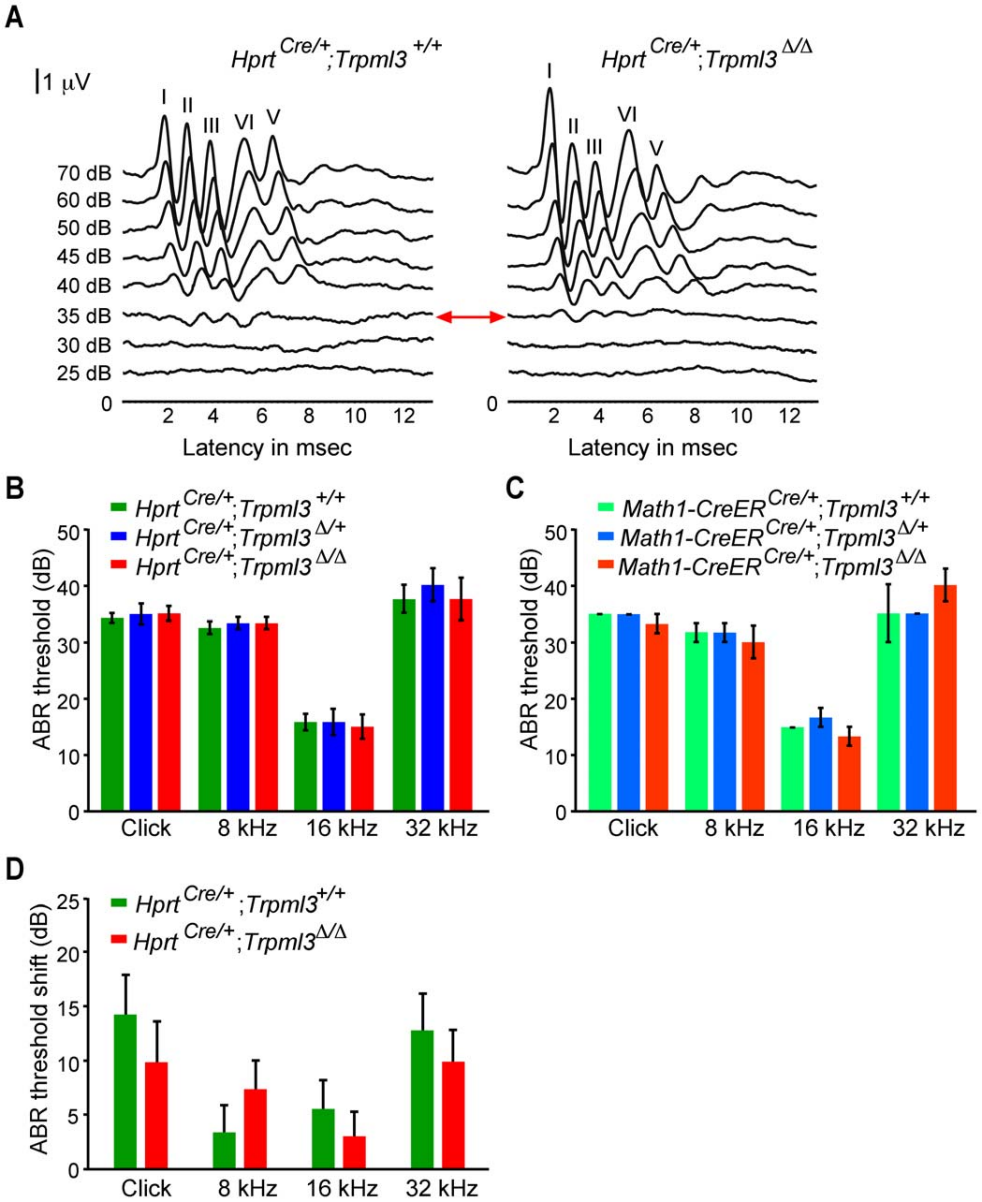
ABR measurements. A, Graph shows representative ABR waveforms of 3-week-old *Hprt^Cre/+^; Trpml3^+/+^* and *Hprt^Cre/+^; Trpml3 ^Δ/Δ^* mice in response to a click stimulus. ABRs were recorded at sound stimulation intensities of 25–70 dB. ABR waves I–V are indicated above the peaks. Red arrow highlights the hearing threshold, which is at 35 dB in this representative example pair. B, Shown are ABR thresholds (mean values ± SEM) to click, 8-, 16-, and 32 kHz stimuli of *Hprt^Cre/+^;Trpml3^+/+^* (n = 6), *Hprt^Cre/+^;Trpml3^Δ/+^* (n = 6), and *Hprt^Cre/+^;Trpml3 ^Δ/Δ^* (n = 6) and C, of *Math1-CreER^Cre/+^;Trpml3^+/+^* (n = 3), *Math1-CreER^Cre/+^;Trpml3^Δ/+^* (n = 3), and *Math1-CreER^Cre/+^;Trpml3 ^Δ/Δ^* (n = 3), respectively. Statistical comparisons of means of different genotypes were made using one-way ANOVA followed by Tukey's post test; p>0.05. D, ABR threshold shifts of 3-month-old *Hprt^Cre/+^;Trpml3^+/+^* (n = 7) and *Hprt^Cre/+^;Trpml3 ^Δ/Δ^* (n = 7) 1 week after the acoustic overexposure of 125 dB SPL at 4 kHz for 4 hr. Shown are mean values ± SEM. No significant differences were observed between genotypes (p>0.05, one-way ANOVA, followed by Tukey's post test).

To investigate whether acoustic challenge of the auditory system would reveal a more subtle role of TRPML3, we exposed *Hprt^Cre/+^*;*Trpml3^+/+^* and *Hprt^Cre/+^*;*Trpml3 ^Δ/Δ^* mice for 4 hr to 4 kHz pure tone at 125 dB SPL [25], [26]. We compared ABR thresholds of littermates of both genotypes before and one week after the noise exposure, and we detected no difference in noise susceptibility between the two groups (Fig. 4D). Unlike knockout of TRPV4, an ion channel expressed by cochlear hair cells, spiral ganglion neurons, and stria vascularis marginal cells [27], which displays increased susceptibility to acoustic injury [25], mice carrying two inactive *Trpml3* alleles did not show increased acoustic vulnerability.

**Figure 5:**
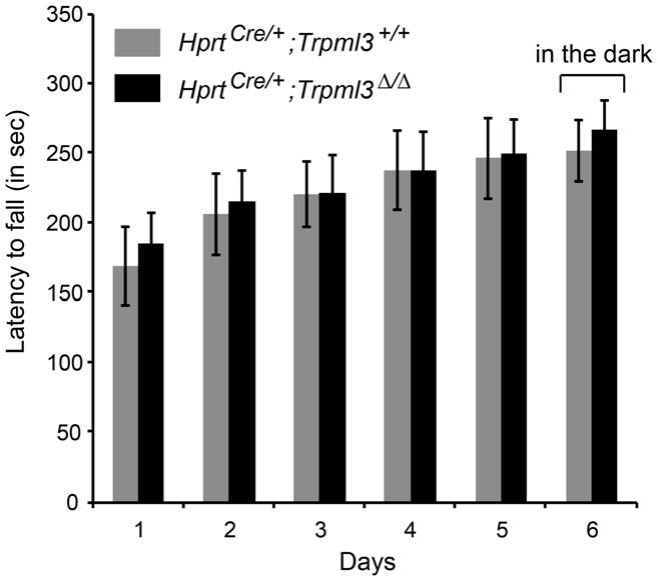
Rotarod experiments. The average latencies to fall ± SEM (in sec) are shown for 3-month-old *Hprt^Cre/+^;Trpml3^+/+^* (n = 8) and *Hprt^Cre/+^;Trpml3 ^Δ/Δ^* (n = 8) mice. The experiment was executed over a time range of 6 days. The day 6 experiment was performed in the dark to exclude compensation via visual cues. The difference between genotypes was not statistically significant at any given time point (p>0.05, one-way ANOVA, followed by Tukey's post test).

Besides evaluating the auditory system, we also assessed vestibular function in *Hprt^Cre/+^*;*Trpml3^+/+^* and *Hprt^Cre/+^*;*Trpml3 ^Δ/Δ^* mice. We did not notice circling behavior, head-bobbing, waddling, or imbalance when walking or when walking along the top of the 3 mm thin cage wall, suggesting normal vestibular function (data not shown). To obtain more objective and quantitative data on potential balance deficits, we performed Rotarod tests [28]. We did not measure a significant difference between *Hprt^Cre/+^*;*Trpml3^+/+^* and *Hprt^Cre/+^*;*Trpml3 ^Δ/Δ^* mice for the 6-day-period tested (Fig. 5), indicating that inactivation of *Trpml3* does not lead to balance defects in this test.

Despite the strong *Va* phenotype, which is caused by constitutively active TRPML3, no overt inner ear phenotype was detectable in mice with ubiquitous *Trpml3* inactivation or when the gene was inactivated from P0–P3 onwards. This finding supports the previous conclusion that the TRPML3(A419P) mutation of *Va* mice is a gain-of-function mutation, which has been hypothesized to cause Ca^2+^-loading of cells and subsequently apoptotic cell death [7]–[11]. Loss of functional *Trpml3*, on the other hand, is not causing a detectable phenotype in the inner ear. Moreover, *Hprt^Cre/+^*;*Trpml3 ^Δ/Δ^* mice were indistinguishable from their wild-type littermates in size and weight. *Trpml3 ^Δ/Δ^* mice inherited the mutation stably and homozygous mutant animals displayed normal breeding performance.

We suggest several possible explanations for the lack of a phenotype in *Trpml3 ^Δ/Δ^* mice. The first possibility is that *Trpml3* is not essential for inner ear function and that in *Va* mice, the channel protein is turned into a constitutively active and cytotoxic channel. This would mean, however, that the native TRPML3 channel does not fulfill essential detectable functions in the inner ear *in vivo*. Another possibility is that our tests were not sufficient to reveal more subtle roles of TRPML3 such as potential modulatory roles in hair cells or other parts of the auditory and vestibular system.

Finally, it is a distinct possibility that native TRPML3 proteins are dispensable subunits of heteromeric channels. A different TRP channel, such as the related TRPML2 might compensate for loss of TRPML3. Heteromerisation of TRPML channels has been shown previously [12], [15]. This hypothesis is supported by the observation that a dominant negative isoform of TRPML3, when transfected into epidermal melanocytes, is able to inhibit activation of a presumptively heteromeric channel consisting of TRPML3 and other unknown subunits [13]. Such a mechanism is potentially testable by generation of mice with inactivating mutations in *Trpml3* and one or more additional genes that encode potential heteromeric subunits. This strategy might ultimately reveal the physiological function of TRPML3-containing channels in sensory hair cells and other cell types of the body.

## Materials and Methods

### Targeting construct

Genomic 129sv/svj mouse DNA was used for PCR-amplification of DNA sequences flanking exon 11 of *Trpml3* (Fig. 1). pBluescript II SK(+) vector (Stratagene): Left arm (*Sal*I), FRT-flanked neomycin resistance marker (*neo^R^*) (*Hind*III), *loxP*-flanked Intron-Exon11-Intron (*Eco*RI), right arm (*Sma*I), and Diphteria toxin A (DTA) cassette (*Spe*I and *Sac*I). The sequence-verified and linearized targeting vector DNA was electroporated into 129sv/svj ES cells (Stanford transgenic research center (http://med.stanford.edu/transgenic)) and two independent clones with proper homologous recombination were selected by PCR amplification and sequencing of the integration sites. The *neo^R^*-cassette was excised with Flp recombinase and the ES cells carrying the targeted *Trpml3* allele were injected into blastocysts to generate chimeras. Mating of male chimeras with C57BL/6J females produced heterozygous transgenic founders that were crossed and maintained in FVB/NJ background, which was selected for analysis because this strain does not exhibit early onset age-related hearing loss [29].

### Mice and Genotyping

FVB/NJ mice (Jackson Laboratory stock number 001800), *Math1*-*Cre*ER™ mice (provided by Dr. S.J. Baker, St. Jude, Memphis, TN), and *Hprt^Cre^* mice (provided by Dr. M. Krasnow, Stanford University) were used. Both driver strains were crossed for at least ten generations into FVB/NJ background before crossing them with mice carrying the *Trpml3^lox^* allele. *Trpml3^lox^* mice had been crossed into FVB/NJ background for at least three generations. Genomic DNA was isolated from mouse tails (DNEasy, Qiagen, Valencia, CA). Animal studies were conducted in accordance with protocols approved by the Administrative Panel on Laboratory Animal Care at Stanford University. The protocol number is #11961.

### Induction of Cre activity

Tamoxifen (Sigma) was dissolved in prewarmed sterile corn oil (Sigma) at a concentration of 3 mg/ml. A 26-gauge needle insulin syringe was used for intraperitoneal injections between P0 and P3 at 3–4 mg/40 g body weight. Three injections were separated by 24 hours.

### Heterologous expression of TRPML3 isoforms and calcium imaging

HEK293 cells were grown and maintained in a standard humidified 37°C incubator, with 95% air and 5% CO_2_. The cells were maintained in DMEM (Cellgro), supplemented with 10% fetal bovine serum (Omega scientific), and 100 µg/ml penicillin and streptomycin (Cellgro). For calcium imaging experiments and localization studies all plasmid constructs were transiently expressed in HEK293 cells with the use of Genejammer (Stratagene), and analyzed 24 hr after transfection. Expression vectors were based on pcDNA3.1 (Invitrogen). For calcium imaging experiments, the cells were loaded with 4 µM fura-2-AM (Invitrogen) in a solution containing 138 mM NaCl, 6 mM KCl, 2 mM MgCl_2_, 2 mM CaCl_2_, 10 mM HEPES, and 5.5 mM D-glucose (300 mOsmol/kg and adjusted to pH 7.4 with NaOH). Measurements of [Ca^2+^]_i_ with the fluorescent indicator were performed using a monochromatic-based imaging system (iMIC platform and Polychrome V monochromator, TILL Photonics). TRPML3 activator SN-2 (5-mesityl-3-oxa-4-azatricyclo[5.2.1.02,6]dec-4-ene) (Specs) was used at a final concentration of 10 µM [13].

### Auditory brainstem response (ABR) measurements and noise exposure

The ABR procedure was done as previously described [11]. The absolute threshold was obtained for each animal by reducing the stimulus intensity in 5 dB steps to identify the lowest intensity at which the first ABR wave was detectable. The latency (time delay from when the stimulus is presented (0 msec) until its peak occurs) was determined after detecting all peaks in click ABR waveforms I–V at 70 dB. Interwave latencies between the peaks of ABR waves I and III were calculated. Three months old *Hprt^Cre/+^*;*Trpml3^+/+^* and *Hprt^Cre/+^*;*Trpml3 ^Δ/Δ^* mice were exposed 4 hr to 4 kHz pure tone at 125 dB SPL (sound pressure level) in a truncated pyramid-shaped exposure box [26] to examine the effects of TRPML3 inactivation on acoustic injury of the cochlea. Shifts in the ABR thresholds were determined after 1 week.

### Motor coordination tests

A four track Rotarod (Columbus Instruments) was used to test for a balance or motoric impairment in 3 months old *Hprt^Cre/+^*;*Trpml3^+/+^* and *Hprt^Cre/+^*;*Trpml3 ^Δ/Δ^*. The mice were acquainted with the instrument (without rotating) daily for five days, and one day before the first trial, the mice were placed on the Rotarod for 90 sec at a constant speed of 3 rpm to familiarize them with the procedure. For testing, the mice were placed onto the rod for a 330 sec trial with constant acceleration from 3 rpm to 40 rpm. Four trials with 45 min inter-trial periods were performed each day. The trials on day 6 were performed in the dark. The time until fall was automatically recorded, while the maximal time was noted for mice staying until 40 rpm.

### Statistical analysis

Data are presented as mean ± SEM with the number of independent experiments indicated (n). Statistical comparisons were made using either one-way ANOVA followed by Tukey's post test or Student's *t* test (unpaired) and KaleidaGraph Synergy software. Differences were considered significant when p<0.01 (*), and highly significant when p<0.0001 (***).
